# 以噬血细胞综合征为首发表现的EB病毒阳性淋巴结T/NK细胞淋巴瘤1例报告并文献复习

**DOI:** 10.3760/cma.j.cn121090-20250922-00431

**Published:** 2026-04

**Authors:** 婧妍 邱, 燕平 刘, 红岭 秘, 益莲 杨, 飞 梁, 慧子 方, 茜婷 贾, 建勇 李, 祎 缪

**Affiliations:** 1 南京市浦口人民医院，东南大学附属两江医院，南京 211800 Nanjing Pukou People's Hospital, Liangjiang Hospital, Southeast University, Nanjing 211800, China; 2 南京医科大学第一附属医院，江苏省人民医院血液科，淋巴瘤中心，南京 210029 Department of Hematology, Lymphoma Center, Jiangsu Provincial People's Hospital, The First Affiliated Hospital of Nanjing Medical University, Nanjing 210029, China

## Abstract

为增进对EB病毒（EBV）阳性淋巴结T/NK细胞淋巴瘤的认识，提升以噬血细胞综合征（HLH）为首发表现患者的早期识别与诊断能力，本文回顾性总结1例以HLH起病的EBV阳性淋巴结T/NK细胞淋巴瘤患者的临床资料，并结合相关文献进行复习。患者男性，58岁，因“发热6 d”就诊，首发临床表现为HLH，虽经积极规范治疗，最终仍于确诊后不足2个月死亡。EBV阳性淋巴结T/NK细胞淋巴瘤临床罕见，易误诊，多数患者确诊时已属晚期，目前尚无标准治疗方案，预后极差。

## Abstract (en)

To enhance our understanding of Epstein-Barr virus（EBV）-positive nodal T/natural killer（NK）cell lymphoma and improve the early recognition and diagnosis of patients presenting with hemophagocytic syndrome（HLH）as the initial manifestation, this article retrospectively summarizes clinical data for a case of EBV-positive nodal T/NK cell lymphoma presenting with HLH at onset and reviews relevant literature. The patient, a 58-year-old male, presented with a “fever for 6 days” as the chief complaint, with the initial clinical manifestation being HLH. Despite active standard treatment, the patient died less than 2 months after diagnosis. EBV-positive nodal T/NK cell lymphoma is clinically rare and easily misdiagnosed. Most patients are diagnosed at an advanced stage. Furthermore, there is currently no standard treatment, leading to an extremely poor prognosis.

EB病毒（EBV）阳性淋巴结T/NK细胞淋巴瘤（EBV-nTNKL）是一种罕见疾病，在2016年世界卫生组织（WHO）分类中被列为外周T细胞淋巴瘤-非特指型（PTCL-NOS）的一个亚型[1]。由于其具备独特的临床病理学及遗传学特征，明显区别于PTCL-NOS和结外NK/T细胞淋巴瘤（ENKTL），2022年第5版WHO分类已将其确立为独立疾病类型[2]。该病好发于东亚人群，多见于老年人，男女发病率之比为（1.3～3.8）∶1。患者典型临床表现包括淋巴结肿大，可合并结外侵犯（不伴鼻腔受累），常伴有B症状。多数患者在确诊时已处于疾病晚期，与PTCL-NOS和ENKTL相比预后更差[3]–[6]。本例患者诊断为EBV-nTNKL，临床首发表现为噬血细胞综合征（HLH），在及时积极的治疗后仍于确诊后不足2个月死亡，现对该患者的临床资料进行报道并进行文献复习。

## 病例资料

患者，男，58岁，受凉后出现发热，伴咽痛、寒战，最高体温39 °C，自服奥司他韦未见好转，于2025年3月17日因“发热6 d”就诊于南京市浦口人民医院呼吸科。无既往史及家族遗传病史。查体：颈部双侧、双侧腹股沟可触及散在肿大淋巴结，较大者位于颈部右侧约4 cm×2 cm，脾肋下约3 cm。入院生化检查：ALT 177 U/L、AST 268.7 U/L。颈部超声及腹腔CT均提示多发肿大淋巴结、脾肿大，患者于2025年3月20日转入血液科后行PET-CT结果示：颈部双侧、左侧锁骨下、纵隔气管旁隆突下、心膈角区、肝胃间隙、脾胃间隙、腹膜后、腹主动脉旁、网膜囊区、肠系膜区、盆腔内、两侧盆壁、两侧髂内外血管旁多发肿大淋巴结，较大者约3.8 cm×2.1 cm，^18^F-氟代脱氧葡萄糖（^18^F-FDG）代谢异常增高（SUVmax 14.7）；脾大，^18^F-FDG代谢弥漫性增高，L1椎体偏左见直径约1.2 cm结节状^18^F-FDG代谢增高灶（SUVmax 5.4），考虑淋巴瘤多发淋巴结、脾浸润，骨浸润可能。2025年3月20日行腹腔淋巴结穿刺，次日行骨髓穿刺。患者入院后三系血细胞进行性下降（最低值：ANC 1.66×10^9^/L、HGB 86 g/L、PLT 21×10^9^/L），纤维蛋白原进行性下降（最低值：0.58 g/L），生化检查：ALT 206.9 U/L、AST 206.0 U/L、总胆红素65.71 µmol/L、甘油三酯2.29 mmol/L、白蛋白26.6 g/L、β_2_微球蛋白7.64 mg/L；LDH 849 U/L（正常参考范围：120～250 U/L）；血清铁蛋白3 404 ng/ml（正常参考范围：30～400 ng/ml）；可溶性CD25（sCD25）125 893.8 pg/ml（正常参考范围：400～2 500 pg/ml），全血EBV：7.14×10^5^拷贝数/ml；患者发热、脾大、全血血细胞减少、纤维蛋白原减低、铁蛋白以及sCD25明显升高，符合HLH的诊断标准，2025年3月22日起给予DEP（脂质体多柔比星、依托泊苷、甲泼尼龙）+培门冬酶方案治疗。

2025年3月24日腹腔淋巴结穿刺病理回报：符合EBV-nTNKL形态学及免疫表型。HE染色：镜下见多形性淋巴细胞弥漫分布，部分胞体较大、核形不规则可见核仁，背景散在凋亡小体（图1A）。免疫组化结果：CD20（+）、PAX5（−）、CD3（+）（图1B）、CD5（−）、CD4（−）、CD8（+）（图1C）、CD2（−）、CD7（+）、CD56（−）（图1D）、TIA1（+）、GRANB（+）、PERFORIN（−）、CD21（−）、CYCLIND1（−）、P53（突变型+）、CD30（−）、ALK（−）、BLC2（−）、BLC6（−）、CD10（−）、MUM1（−）、TDT（−）、C-MYC（−）、Ki-67（75％+）（图1E）；原位杂交：EBV编码的小RNA（EBER）（+）（图1F）。淋巴结穿刺物涂片：可见部分淋巴细胞体积偏大，胞质量丰富，染淡蓝色较细致，可见核仁，易见核分裂象。淋巴结流式细胞术免疫分型：异常T细胞占所有细胞的71.3％，表达特点：CD3^+^CD8^+^CD2^+^CD7^+^TCRab^+^CD45RO^+^CD4⁻CD5⁻CD45RA⁻CD57⁻CD56⁻CD16⁻TCRrd⁻CD10⁻。2025年3月25日骨髓涂片形态：粒系、巨核系增生活跃，红系增生减低，血小板成簇可见；见噬血组织细胞。骨髓活检：见噬血组织细胞。骨髓流式细胞术免疫分型：克隆性T细胞占2％，其中CD3^+^CD8^+^TRBC1^+^T细胞<15％。骨髓细胞染色体核型分析：46,XY[20]。骨髓细胞二代测序：DNMT3A p.Gly543Cys，变异等位基因频率为48.6％；TET2 p.Gln116fs，变异等位基因频率为49.1％。综上诊断：EBV-nTNKL（Ann Arbor Ⅳ期B组，体能状态评分为2分，IPI评分为4分，PIT评分为3分）。

**图1:**
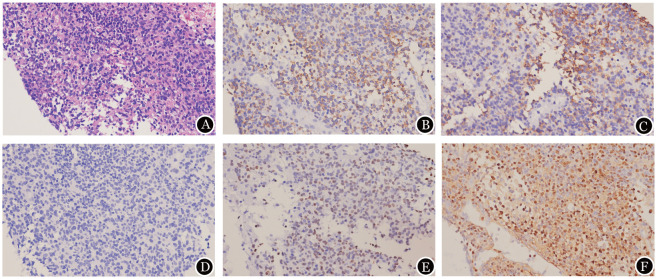
EB病毒阳性淋巴结T/NK细胞淋巴瘤患者淋巴结穿刺组织HE染色（A，×400）及免疫组化结果（B-F，×400） **A** 淋巴细胞体积偏大，胞质量丰富，染淡蓝色较细致，可见核仁；**B** 肿瘤细胞CD3阳性；**C** 肿瘤细胞CD8阳性；**D** 肿瘤细胞CD56阴性；**E** Ki-67 75％阳性；**F** EB病毒编码的小RNA（EBER）阳性

2025年3月26日患者转至江苏省人民医院进一步治疗，行病理会诊：符合EBV阳性T/NK淋巴组织增殖症，免疫组化示CD3^+^CD5⁻CD8^+^CD4⁻CD56⁻TIA1^+^GRANB⁻PERFORIN⁻；鉴于PET-CT影像学评估所示全身多发淋巴结肿大，淋巴结组织TCR基因重排阳性，本例符合EBV-nTNKL。最终患者诊断为：EBV-nTNKL，并于江苏省人民医院行后续治疗，治疗上加用塞利尼索，患者应用DEP+培门冬酶+塞利尼索治疗后有好转，2025年4月14日患者再次发生HLH，予以CHOP（环磷酰胺+脂质体多柔比星+长春地辛+泼尼松）方案联合塞利尼索治疗，2025年5月2日患者再次出现高热，HLH相关指标急剧升高，予以脂质体米托蒽醌+依托泊苷+替雷利珠单抗+戈利昔替尼治疗。患者最终因疾病控制不佳，在诊断后第55天放弃治疗，于院外死亡。

## 讨论及文献复习

本例患者以HLH起病，即持续高热、进行性全血细胞减少、纤维蛋白原降低、脾大、铁蛋白及sCD25显著升高、骨髓中可见噬血现象，符合HLH-2004诊断标准。本例经淋巴结穿刺活检确诊为EBV-nTNKL。鉴别诊断方面需要与EBV阳性T/NK细胞增殖性疾病谱系相鉴别，如ENKTL、慢性活动性EBV感染（CAEBV）、儿童系统性EBV阳性T细胞淋巴瘤，以及EBV阳性血管免疫母细胞性T细胞淋巴瘤（AITL）等[2],[7]，结合本例患者成人发病、无CAEBV病史，淋巴结受累、无鼻腔及皮肤等结外病灶，免疫表型示CD3^+^CD5⁻ CD8^+^CD4⁻CD56⁻TIA1^+^EBER^+^及TCR基因重排阳性，EBV感染肿瘤细胞且肿瘤细胞为CD8阳性T细胞，最终将其诊断为EBV-nTNKL。治疗方面，该患者以噬血表现起病，EBV负荷较高，使用DEP方案联合培门冬酶同时进行EBV相关噬血与淋巴瘤相关噬血的治疗。

目前，EBV-nTNKL临床资料主要来源于个案及小宗病例分析，其中合并HLH者罕见，该病合并HLH发生率为6.7％～53.8％[3]–[4],[8]–[9]。尽管此类病例少见且缺乏大样本数据，但结合本例及有限文献报道，仍可发现一些共性的临床挑战。文献[3]报道的1例及文献[9]报道的14例患者均表现为爆发性的临床进程和极差预后，本例与多数报道病例的中位生存期仅约0.75个月，提示合并HLH是极端不良的预后指标。因此，对于确诊EBV阳性T/NK细胞淋巴瘤的患者，建议常规并动态监测HLH相关指标，以实现HLH的早期识别及干预。

EBV-nTNKL的病理形态学通常表现为中等大小的淋巴细胞，其典型肿瘤细胞与弥漫大B细胞淋巴瘤的中心母细胞具有相似的形态特征。本例患者的淋巴结穿刺涂片细胞学及组织HE染色显示，肿瘤细胞符合上述中心母细胞的典型形态学特点，与Nicolae等[10]的报道一致。此外，该患者的淋巴结免疫组化示CD3^+^CD8^+^CD56⁻EBER^+^且流式细胞术证实细胞表面CD3阳性，支持T细胞来源，这也与目前研究一致，研究表明EBV-nTNKL中NK细胞来源的比例不足20％[3]–[4],[8],[11]。Kato等[12]对EBV进行的全基因组测序分析发现，nTNKL病例中检出的EBV毒株与当地其他疾病相关的EBV基因组聚类良好，并未发现特异性存在于EBV-nPTCL中的独特病毒株。

TET2和DNMT3A是调控表观遗传的关键基因，其突变常见于急性髓系白血病（AML）和骨髓增生异常综合征（MDS），在T细胞淋巴瘤（特别是AITL亚型）中也较为多见[13]。Kato等[12]研究表明，在EBV-nTNKL患者中，TET2和DNMT3A突变的发生率分别可达68％和32％，且所有DNMT3A突变都伴随TET2突变。同时发现这些共存突变往往与潜能未定克隆性造血（CHIP）相关，提示该类淋巴瘤可能起源于已携带突变的造血干/祖细胞。值得注意的是，DNMT3A突变在AML中已是明确的不良预后指标，而在EBV-nTNKL中具有TET2/DNMT3A突变患者预后较差[12]。本例同时检出这两种突变，预后差，与上述研究一致。两种基因突变在血液系统恶性肿瘤中是否具有普遍的临床预后价值需进一步探索。

EBV-nTNKL具有高度侵袭性，常表现为对传统化疗耐药。CHOP样方案疗效有限，患者预后较差[4]。目前临床更多推荐使用含左旋门冬酰胺酶的方案（如SMILE方案）作为一线治疗[14]。在免疫微环境方面，EBV-nTNKL中PD-L1表达常上调[11]，提示PD-1抑制剂可能具有一定治疗潜力。但需注意的是，伴有TET2突变的患者PD-L1表达往往较低[12]，且使用免疫检查点抑制剂存在诱发或加重细胞因子风暴的风险，可能加剧HLH相关的免疫过度激活。TET2和DNMT3A突变通常提示预后不良，为靶向治疗提供方向。去甲基化药物（如地西他滨、阿扎胞苷）在复发/难治性AITL和PTCL-NOS中显示一定疗效[15]–[16]。因此，对伴有此类突变的EBV-nTNKL患者，采用含去甲基化药物的治疗方案是值得探索的策略。

在常规治疗效果不佳的情况下，细胞免疫治疗显示出重要潜力。EBV特异性细胞毒性T淋巴细胞（EBV-CTL）在EBV相关疾病中能够有效降低病毒载量、改善临床症状，并可作为完全缓解后的维持治疗手段[17]。另一方面，嵌合抗原受体T（CAR-T）细胞疗法在T细胞恶性肿瘤中也取得进展。除靶向自身抗原（如CD7、CD5）的CAR-T细胞疗法外，针对EBV潜伏膜蛋白2A（LMP2A）的CAR-T细胞疗法提供了新思路。该策略能精准清除EBV感染细胞，并规避靶向自身抗原导致的自相杀伤（fratricide）及继发性免疫缺陷风险[18]。未来，通过开发双靶点CAR、联合检查点抑制剂等策略，有望进一步提升其疗效，为此类预后极差的患者带来新的希望。

异基因造血干细胞移植（allo-HSCT）是患者目前唯一可能实现长期生存乃至治愈的手段。个案报道显示部分患者接受allo-HSCT后获得长期无病生存[9],[19]。然而，由于例数有限，其最佳适应证和移植时机仍需进一步明确。综合现有证据，对于这类侵袭性强、常规疗效差的患者，在通过新型疗法（如EBV-CTL、CAR-T细胞或去甲基化药物）获得疾病控制后，及时桥接allo-HSCT，可能是目前最具合理性的治疗路径。

本研究具有一定的局限性：个案报道代表性有限，患者病程中虽先后尝试DEP+培门冬酶+塞利尼索、CHOP方案联合塞利尼索等多线治疗方案，但病情仍控制不佳，患者于诊断后第55天放弃治疗死亡，提示EBV-nTNKL合并HLH对现有多种治疗策略均难以实现持久缓解，预后极差；本例虽检出TET2/DNMT3A突变，但未能深入探讨其与免疫微环境的关联。

总之，EBV-nTNKL是一种在诊断、临床管理和治疗策略方面均存在挑战的侵袭性疾病，未来需积累更多病例，进一步研究以建立针对性的治疗策略。
